# Dr. Harry Lane

**Published:** 1917-07

**Authors:** 


**Dr. Harry Lane,** Willamette University, Salem, Oregon, 1876, died at San Francisco, May 25, aged 62. He was born in Washington and was U. S. Senator from 1913 till his death.

